# Something for the Consideration of Faculties

**Published:** 1897-06

**Authors:** 


### SOMETHING FOR THE CONSIDERATION OF FACULTIES.

INFORMATION has been placed in the hands of the editors of the JOURNAL, bearing upon the granting of diplomas to several students during '97, in which a very grave question arises as to how much actual time had been spent in the college granting the diploma, and how much time should have been allowed for the time spent at other colleges. We believe that this matter of allowance of time should be one of the most important points to be taken up at the next meeting of the Association of Faculties; and some fixed, definite plan should be devised by which schools should be guided in receiving their students and in granting them an allowance for time spent in other colleges. Much criticism is being indulged in, much surprise expressed, many inquiries are being made, bearing upon these points, and, unless the Association of Faculties can arrive at an understanding, there is danger in this direction that promises to retard the advance of higher veterinary education.

